# Factors Affecting Length of Inpatient Forensic Stay: Retrospective Study From Czechia

**DOI:** 10.3389/fpsyt.2022.825615

**Published:** 2022-05-04

**Authors:** Marek Páv, Martina Vňuková, Ivan Sebalo

**Affiliations:** ^1^Psychiatric Hospital Bohnice, Prague, Czechia; ^2^Department of Psychiatry, First Faculty of Medicine, Charles University and General University Hospital, Prague, Czechia; ^3^School of Psychology and Computer Science, University of Central Lancashire, Preston, United Kingdom; ^4^Ashworth Research Centre, Ashworth High Secure Hospital, Mersey Care NHS Foundation Trust, Liverpool, United Kingdom

**Keywords:** forensic treatment, length of stay, psychosocial needs, HoNOS, HoNOS secure, unmet needs for care

## Abstract

**Objectives:**

The length of forensic stay (LoS) is a subject to country-specific legal and service systems. Therefore, the identification of common factors targetable by treatment is at the forefront of forensic psychiatric research. In this study, we present the first reports of forensic characteristics of patients from the Czechia.

**Methods:**

We conducted a retrospective analysis of data from 260 inpatients discharged from the Bohnice Hospital (Prague) and obtained a set of sociodemographic and clinical variables as well as the Health of the Nation Outcome Scale (HoNOS) and HoNOS-secure scores.

**Results:**

The following variables were identified as significantly associated with a longer LoS: older age, length of previous psychiatric hospitalization, olanzapine equivalent, clozapine treatment, psychosocial dysfunction, psychotic or paraphilic disorder diagnosis, and sexual offense. A shorter LoS was associated with being in a relationship, being employed before hospitalization, receiving personal support, and committing an index offense under the influence of substance. While the HoNOS score and HoNOS symptom subscale predicted a longer LoS, the HoNOS-secure subscale predicted a shorter stay.

**Conclusion:**

In the European context, our hospital has a relatively low LoS. The results are consistent with findings linking psychotic disorders and paraphilia with a longer LoS in forensic treatment. Higher doses of antipsychotic medication or clozapine prescriptions were associated with a longer LoS. The results show a high level of unmet needs in this population, highlighting the importance of the availability of follow-up service.

## Introduction

According to Czech law, protective treatment (PT) can be imposed as a criminal sanction. The necessary prerequisite is a criminal offense by a person with a mental disorder or illness that diminishes his/her criminal responsibility (1). These patients are then treated with an aim to improve their mental health condition and concurrently protect the public from possible imminent harm. In some cases, treatment requires extended time as there is a public safety concern when recidivism risk is not sufficiently reduced. Increased attention is drawn to PT prolongation because disproportionately extended stays in forensic facilities can lead to human rights violations and the lack of respect for patients’ autonomy (2). The legal requirement of “proportionality” of PT as a criminal sanction is contained in European and Czech law (3, 4). The cause for the prolongation of PT are unknown because PT duration is more or less stable in some countries (5, 6). Interstate differences might be due to differences in legislation, processes, court trial procedures, or criminal policy (3, 7). The service system setting and seemingly unrelated factors, such as the global democracy index, gross domestic product, and total healthcare expenditures, may also play a role (5).

In the search for a balance between the adequate length of PT and the severity of the clinical condition, experts have tried to identify the key factors underlying the treatment duration. Several studies have explored the sociodemographic and treatment-related characteristics associated with length of forensic stay (LoS), and while some are consistently replicated others are not. Sociodemographic predictors of a longer LoS are male sex, an early manifestation of offending behavior, committing multiple offenses over an extended period, and the severity of offending behavior (8–14). However, the relationship between offense severity and prolonged LoS has not been consistently replicated (8). Clinical parameters predictive of longer LoS include previous psychiatric admissions, diagnosis of schizophrenia or other psychotic vulnerability, resistance to treatment or persistence of symptoms, and committing an offense as a result of hallucinations or during discontinuation of drug treatment (8–11). In the treatment, a longer stay is predicted by a history of absconding, slow treatment progress, complex mental health problems, or severe assault on staff (15). In a previous Czech study, we compared differences between long-stay forensic inpatients and discharged patients and concluded that committing more than one violent crime in the past, lacking insights into the illness, and being uncompliant with the treatment regimen are significantly associated with continued hospitalization (16).

Legal procedures can confound these variables as conditional release or the termination of inpatient treatment require judicial approval (17). However, legal considerations often constitute factors that cannot be targeted by the therapeutic approach; therefore, these results need to be interpreted with caution and focus should shift away from legal considerations [Kirchebner et al. (18)]. In addition, the arrangement or availability of follow-up services also affects LoS (12, 13). Finally, some studies have explored the external factors associated with LOSs, and a large-scale study of 16 European countries identified four major themes concerning external factors. The factors are care and treatment pathways, resources, legal and systemic impacts, and shared expertise (19).

The use of complex tools or outcome measures, such as Camberwell Assessment of Need Forensic Version (CANFOR) or the Health of the Nation Outcome Scale (HoNOS), enhances output comparability and focuses on critical areas of needs and risks (20). Unmet needs may hinder patients’ treatment progress; therefore, routine monitoring of needs can aid resource allocation and facilitate benchmarking (21).

Currently, forensic population data from Eastern European countries, including the Czechia, are scarce. Currently, there are 13 hospitals in the Czechia providing forensic inpatient treatment to 950 patients and treating approximately 2,300 patients in 386 outpatient clinics. The Czech forensic system also includes two secure detention facilities (85 patients). Czech psychiatric care is currently undergoing a deinstitutionalization reform process, with the aim of establishing forensic multidisciplinary teams to provide the missing link in the treatment process. Certain studies, such as the one by Páv et al. (1), provide details about the current state and procedures of forensic treatment in the Czechia.

The main aim of this study was to identify critical sociodemographic and treatment-related factors associated with LoS. In this study, we hypothesized that a diagnosis of severe mental illness would be significantly associated with a longer LoS. Furthermore, the current study investigated the ability of the Czech translation of HoNOS-secure to monitor the forensic inpatient population’s mental health and security needs.

## Materials and Methods

### Data Collection

This study was conducted at the Bohnice Hospital (Prague, Czechia). This hospital is responsible for inpatient PT of all types. The catchment area is approximately 1.2 miles, which covers 9% of the population and is demographically representative of the Czech patient population with PT (1). The hospital offers forensic care in 36 medium secure beds, 35 low secure beds, and 18 low secure beds in a specialized ward with a sex-offender treatment program.

Our study group included 260 inpatients discharged from inpatient PT from January 1, 2015 to June 30, 2020.

A trained evaluator assessed security needs using the HoNOS-secure (22). The seven-item secure subscale complements the original HoNOS scale, allowing the tracking of clinical progress within a secure setting against a range of mental health needs (20, 23). The rating is based on the current need for safe care, taking into account past behavior, attitudes, treatment progress, and treatment prospects (24). This scale was translated into the Czech language by one of our team members, and then a back-to-back translation was performed to ensure accuracy. During the process, we ensured that the vocabulary was kept in line with the HoNOS scale that has been previously used in the Czechia. The evaluator was trained based on the methodology recommended in the HoNOS-secure guide, and further advice was provided by evaluators who were previously trained in the HoNOS scale by the Ministry of Health.

Furthermore, the HoNOS, which is composed of 12 scales, is used (25) to measure behavior, impairment symptoms, and social functioning in adults. The HoNOS is used in the Czechia as a standard clinician tool to assess the needs of inpatients with severe mental illness and is considered an effective way of measuring the patient’s state. As part of the deinstitutionalization project operating within psychiatry reform, each hospital has trained clinicians on the HoNOS scale.

Lastly, we collected the information on medication dosages from medical records. The classical mean dose method was used to calculate the olanzapine equivalent of other antipsychotic doses of medication treatment to make the data comparable (26, 27).

Based on the literature review, we defined variables and clinical factors impacting LoS as follows: (1) personal variables, such as legal status, school and professional qualifications, work, and marital history; (2) clinical assessment data, such as mental health and insight; and (3) legal criminal data, such as convictions before admission to a forensic psychiatric hospital, type of index offense. Data were extracted using all available information sources, including electronic hospital records, expert opinions in medical records, and other health notes included in patients’ files. An independent researcher performed data reliability check during the data collection process by extracting five randomly chosen sample cases.

### Ethics

The Ethics Committee at the Bohnice Psychiatric Hospital approved the study.

### Statistical Analysis

The analysis was performed using R software version 4.0.3 3 (28). There were numerous categorical and continuous variables with non-normal distributions. Thus, this procedure precluded the creation of a single correlation matrix that would indicate the variables related to LoS. We first used linear regression for variables organized according to thematic groups (sociodemographic, crime-, and treatment-related) to highlight those included in the final regression model. All analyses were bootstrapped with 5,000 samples to produce bias-corrected 95% confidence intervals (CIs) to ensure that the effects shown to be significant were not spurious (29). Variables that were identified as being associated with the length of stay were added to one model.

## Results

### Sample Characteristics

The study group included 227 men and 33 women. The mean age at discharge was 42.26 years (standard deviation (SD): 12.79 years). The mean LoS was 496.01 days (SD: 698.9 days). Educational level was generally low: 6.9% of the sample achieved a university degree, while 21.53% graduated. Of the samples, 27.69% were in a relationship before hospitalization, 45% were unemployed, 9.2% of patients had a full-time job, 30.7% were living independently, 11% had sheltered housing, 12.3% were staying in a hostel, and 43% had no housing.

In half of our samples, 51.15% of patients underwent psychiatric treatment, 11.53% underwent sex offender treatment, 11.53% underwent substance misuse treatment, and 29.61% underwent a combination of all of the above. The most prevalent diagnostic groups in our sample were patients with substance abuse disorders (74%); 18% of the sample patients were diagnosed with substance use disorder only, while 57% of the sample patients were diagnosed with comorbidities, such as psychotic disorders (25%) and personality disorders (F6 ICD-10 group) (47%). While the average overall HoNOS score was 2.05 (SD = 2.71), the average score on the subscales was lower. The behavioral, impairment, symptom, and social subscales had a mean of 0.25 (SD = 0.77), 0.65 (SD = 1.18), 0.63 (SD = 1.06), and 0.52 (SD = 1.2), respectively.

Regarding the criminal history, 66% of the samples committed at least one violent act, 11% committed sex offenses, and 19% committed property or other crimes. Finally, 38% of the crimes were committed under the influence of psychoactive substances. Concerning treatment pathways, 31% of patients were admitted from imprisonment, 12.3% from outpatient PT, and 12.6% from hospitalization in a general psychiatry ward. The average score on HoNOS-secure was 3.03 (SD = 1.83).

Of the patients, 59.6% were discharged to outpatient PT. In 32.3% of the cases, the treatment was terminated completely (PT was revoked by the court); in 4.6% of the cases, there was a continued PT (and the interruption of this treatment was due to various causes), and in one case, the patient was transferred to the high-security detainment. Overall, the average length of stay of the sample was 496.01 calendar days (SD = 698.97).

### Factors Influencing the Length of Stay

Based on the preliminary regression analyses (fully reported in the Supplementary Appendix Table 1), the following variables were positively associated with longer LoS: being older at discharge; having a longer previous psychiatric ward hospitalization(s); having a psychotic disorder (as F20 diagnosis) or paraphilia, being treatment-resistant [patient is non-responsive to at least two different antipsychotic medications from different groups, in doses equivalent to chlorpromazine 1,000 mg/day for at least 6 weeks (30), generally this is established after reassessment of the diagnosis after excluding all other comorbidities. This diagnosis was established before our study by the patients’ primary physician]; using a higher antipsychotic dose (calculated as olanzapine equivalent), which was associated with LoS alone as well. Committing at least one sex crime, having a social facility or sheltered housing as a housing option after release; lacking personal support (as evidenced by contact with family or a friend during hospitalization), or lacking housing after discharge was also associated with a longer LoS. Being discharged from the open or low-secure ward compared to the medium-secure ward and having higher HoNOS scores were associated with a longer LoS.

The following variables had negative associations with LoS: shorter length of stay: female sex, being married or engaged, having stable employment before hospitalization, having a combined or substance abuse PT, committing an index offense under the influence of substances, being discharged from the medium-security ward, and sex offender treatment program compared to the other wards, and having higher HoNOS-secure scores.

### Unified Model

Based on these regressions, we ran a complete model that included either continuous (e.g., age) or binary (e.g., being diagnosed with a paraphilia disorder) variables that were significantly associated with LoS by both *p* and CI that did not include 0. The final model (Table 1) fitted the data well, *F*(238,10) = 8.52, *p* < 0.001, and explained almost a quarter of the variance in the length of stay (adjusted *R*^2^ = 0.23).

**TABLE 1:** Factors increasing or decreasing the length of stay.

	Estimate	95% CI	Std. Error	*t*-value	*p*-value	Std. Beta
(Intercept)	−76.86	[−713.26, 224.14]	171.35	−0.45	0.65	
**Age**	**9.69**	**[4.32, 20.52]**	**3.22**	**3.00**	**0.003**	**0.18**
Previous hospitalization length (days)	−0.13	[−0.67, 0.29]	0.21	−0.62	0.53	−0.04
**Total Olanzapine Equivalent**	**10.35**	**[1.47, 25.19]**	**3.62**	**2.86**	**0.005**	**0.24**
**Treatment-resistance**	**353.45**	**[78.1, 827.6]**	**117.49**	**3.01**	**0.003**	**0.20**
**HoNOS secure total**	−**50.39**	**[**−**96.97,**−**7.78]**	**24.65**	**−2.04**	**0.042**	**−0.14**
**HoNOS total**	**62.67**	**[11.31, 80.16]**	**19.58**	**3.20**	**0.002**	**0.23**
Psychotic disorders (F2)	−94.89	[−299.39, 112.32]	106.49	−0.89	0.38	−0.07
**Paraphilia disorders (F65)**	**548.07**	**[285.4, 994.1]**	**179.80**	**3.05**	**0.003**	**0.27**
Crime committed under substance(s) (0-no, 1-yes)	47.14	[−70.72, 228.22]	78.61	0.60	0.55	0.04
At least one sexual crime (0-no, 1-yes)	−100.90	[−481.0, 158.1]	176.45	−0.57	0.57	−0.05

*R^2^ = 0.26, Adjusted R^2^ = 0.23, F(238,10) = 8.51, p < 0.001.*

*Values in bold are those with significant effects.*

A paraphilic disorder had the strongest significant positive association with LoS, followed by olanzapine equivalent of antipsychotic medication, total HoNOS score, treatment resistance, and older age. For instance, having the diagnosis of paraphilia disorder increases LoS by 548.07 days when the other factors are held equal. The only variable associated with a shorter LoS was the total HoNOS-secure score. With an increase in the HoNOS-secure by one score, LoS, on average, decreases by 50.39 days. Although the value of *p* for this estimate was borderline, the bootstrapped CI did not include 0, indicating that a negative association between HoNOS-secure and LoS in the hospital may present in the population.

### Health of the Nation Outcome Scale Subscales

To further investigate the relationship between HoNOS and LoS, linear regression with four HoNOS subscales was performed (Table 2). The model was a good fit, *F*(4,255) = 6.53, *p* < 0.001, and the HoNOS Symptom subscale was significantly and positively associated with LoS, meaning that with every additional score there was, on average, a 153.72-day increase in LoS.

**TABLE 2:** Association of Health of the Nation Outcome Scale (HONOS) subscales with the length of stay.

	Estimate	Std. Error	*t*-value	Betas	*p*-value	95% CI
(Intercept)	341.56	54.57	6.26		<0.001	[204.2, 435.5]
HoNOS behavioral	–58.66	56.01	–1.04	–0.06	0.29	[−144.61, 41.87]
HoNOS impairment	56.63	42.78	1.32	0.09	0.18	[−30.90, 197.31]
**HoNOS symptoms**	**153.72**	**40.78**	**3.76**	**0.23**	**<0.001**	**[49.8, 351.3]**
HoNOS social	66.5	43.24	1.53	0.11	0.12	[−40.96, 258.56]

*R^2^ = 0.09, Adjusted R^2^ = 0.07, F(4,255) = 6.53, p < 0.001.*

*Values in bold are those with significant effects.*

Next, the effect of HoNOS subscales was compared between patients diagnosed with psychotic disorder and patients who were either diagnosed with disorders due to substance use or disorders due to substance use and personality disorder, and other participants. This model also fit the data well, *F*(14,245) = 5.92, *p* < 0.001, and explained 20% of the variance in LoS (Table 3).

**TABLE 3:** Effect of interaction between HONOS subscales and diagnosis on the length of stay.

	Estimate	Std. Error	*t*-value	Betas	*p*-value	95% CI
(Intercept)	588.05	104.18	5.64		< 0.001	[425.7, 834.1]
**Psychotic disorder (F2)**	–389.81	142.66	–2.73	0.26	0.006	[−811.6, −68]
**Substance use and PD (F2 only and F2 + F60/F62)**	–328.92	128.05	–2.56	–0.23	0.01	[−586.1, −158.1]
HoNOS behavioral	–58.38	80.47	–0.72	–0.06	0.46	[−222.7, 46.8]
HoNOS impairment	–4.21	68.04	–0.06	–0.007	0.95	[−138.29, 125.90]
HoNOS symptoms	–55.34	92.35	–0.59	–0.08	0.54	[−190.63, 89.75]
HoNOS social	4.76	62.10	0.07	0.008	0.93	[−96.64, 151.45]
Psychotic disorder HoNOS Behavioral	22.31	121.63	0.18	0.01	0.85	[−211.12, 795.11]
Psychotic disorder HONOS Impairment	201.27	97.80	2.05	0.14	0.04	[−42.2, 586.3]
**Psychotic disorder HoNOS symptoms**	**241.44**	**105.74**	**2.28**	**0.26**	**0.02**	**[33.3, 529.9]**
Psychotic disorder HoNOS Social	373.67	99.24	3.76	0.62	<0.001	[−22.7, 827.7]
Substance use and PD HoNOS behavioral	45.93	151.05	0.30	0.06	0.76	[−163.81, 209.23]
Substance use and PD HoNOS impairment	57.66	100.05	0.57	0.09	0.56	[−79.28, 213.64]
Substance use and PD HoNOS symptoms	60.51	132.23	0.45	0.04	0.64	[−102.22, 201.66]
Substance use and PD HoNOS social	66.27	154.65	0.42	0.04	0.66	[−113.02, 412.21]

*R^2^ = 0.25, Adjusted R^2^ = 0.21, F(14,245) = 5.91, p = < 0.001.*

*Values in bold are those with significant effects.*

Patients with low scores on HoNOS subscales and diagnosed with psychotic disorders (as well as those diagnosed with substance use and personality disorder) had lower LoS than those with low HoNOS-secure subscales and different diagnoses. Meanwhile, for those diagnosed with psychotic disorder, each score on the HoNOS symptom subscale accounted for an increase in LoS, on average, by 241.33. Although the interactions between the diagnoses of psychotic disorder and HoNOS impairment subscales as well as between psychotic disorder and HoNOS social subscales were significant, their 95% bootstrapped CI was 0, indicating that the effect was spurious.

## Discussion

The main aim of this retrospective study was to evaluate the associations between variables and LoS and to explore differences in the study group. To our knowledge, this is the second study investigating the forensic population in a large sample in the Czechia, and the results enable the inclusion of Czech data in the international context and the comparison of Czech parameters with other cohorts. We extended the focus of previous studies by comparing discharged patients and those remaining hospitalized in the long-term forensic stay to short-term forensic hospitalizations (16).

Our hospital LoS of 1.36 years places us among European countries, such as Poland, Latvia, or Italy, with the shortest forensic stays in (5). The average LoS of inpatient forensic treatment in Czechia is 2.6 years, and there is a large variation in LoS between hospitals; however, the Bohnice hospital belongs to the facilities with the shortest LoS in the country.

Overall, our findings correspond to the results obtained for general PT samples demonstrating social maladaptation, inability to achieve stable relationships, or employment before inpatient treatment (9, 15). Almost three-quarters of our sample lacked close relationships and had a lower level of formal education, and only one-third lived independently. We contributed to previous evidence of the problematic socioeconomic situation of patients with psychotic illness obtained from a general patient sample in the Czechia (29). The average age of our sample was 42 years, which corresponds to other cohorts (8, 11). Older age was another factor associated with longer LoS in our cohort. The majority of our sample consisted of men (87.3%), which is similar to other European states (10, 31–33). We found that male sex was associated with LoS, replicating previous results (9, 14), while others found no association (11).

Our results align with previous findings demonstrating that the diagnosis of psychotic disorder or schizophrenia and previous psychiatric hospitalization predict a longer LoS (33, 34). In contrast to some studies, we found that alcohol or substance use decreased LoS (13, 34). In our sample, mood disorder was not associated with a longer LoS, which replicates previous results (10, 13). Resistance to antipsychotic treatment (indicated by clozapine prescription) is a fundamental clinical factor increasing LoS in our sample. Our results also show that a higher total antipsychotic dose (calculated in olanzapine equivalent) is predictive of longer LoS. Higher antipsychotic doses may indicate treatment resistance in cases where clozapine is contraindicated. The impact of combined treatment with antipsychotics on LoS has been previously demonstrated (31). The findings from our hospital indicate that psychotic symptoms are the main drivers of assaultive behavior among inpatients with psychosis; therefore, successfully targeting symptoms is essential for violence prevention (35).

Almost two-thirds of our sample were violent offenders, which are consistent with the findings in Sweden (10), Great Britain (9), or Ireland (17). Some findings point to the severity of an index offense as a robust predictor of prolonged LoS (10–12); we could not link serious offenses (murder or attempting it) to prolonged LoS. This could be explained by the fact that only a small minority of our sample committed a severe violent act (*n* = 10 attempted or committed murder), even though the PT of our cohort was sentenced for violent acts in the majority of the cases. Committing at least one sex offense is predictive of longer LoS (12). In contrast, enrollment in the sex offender program was not associated with prolonged LoS in our sample. An explanation could be that perpetrators who cooperate with treatment successfully undergo a treatment program and are conditionally discharged. Those who do not cooperate stay detained for a longer period of time. Committing an index offense under the influence of substances is associated with a shorter LoS, which is in line with other studies (15, 17). This can be explained by a relatively large proportion of patients accomplishing substance abuse programs in a medium-security ward. This finding is supported by the type of treatment-LoS association, substance abuse or combination treatment (substance abuse plus sex-offender or psychiatric patients). Those patients who are sentenced, tend to have shorter LoS than those with psychiatric or sex-offender treatments. Different treatment programs for different patient groups, according to diagnosis, could thus contribute to the difference in LoS (e.g., substance abuse programs run in the medium-secure ward vs. the rehabilitation program in the general ward).

The HoNOS score is a reliable estimate of LoS. Our results show that patients diagnosed with psychotic disorders with higher symptom levels remain hospitalized for more prolonged periods, and are often discharged to social facilities because they lack housing possibilities. One explanation is that prolonged hospitalization in general psychiatry or a low-secure ward is caused primarily by psychosocial barriers rather than by the risks presented by a given patient. When psychosocial disability is an issue, the availability of the following community services affects LoS (12). In addition, having an ongoing close relationship and being employed before admission to forensic psychiatry predicted shorter stays; this is a replication of the previous findings (8, 13).

The counterintuitive finding is an association between higher HoNOS-secure scores and a shorter LoS. These scores were low in comparison with the other cohorts. This tool captures a higher level of forensic needs in substance abuse patients discharged from closed to medium-security wards. Therefore, accomplishing a substance abuse program is a prerequisite for the termination of inpatient treatment if there is no severe mental health issue. In contrast, those with psychiatric treatment programs in low-security wards received lower scores. Thus, the prolonged stay is presumably caused by the unsatisfied complex needs of the group of patients with severe mental illness and rather than the risks they pose to society. We consider the HoNOS-secure suitable for routine use as it is simple to administer and sensitive to change (23).

### Limitations

One of the limitations of our study is its retrospective design. We failed to obtain a comprehensive data set to compare all variables significantly associated with LoS, as studied in previous studies abroad, such as the age of the first offense or in-treatment behavior including an absconding history of comprehensive aggressive incidents that occurred in the past.

## Conclusion

Several conclusions can be drawn from these findings. First, patients who stay relatively longer in treatment often have a greater psychotic vulnerability, a history of psychiatric ward hospitalization before PT, and have employment and housing problems before hospitalization. It is essential to build community services that can meet complex social needs. A second group of patients with longer LoS are formed by those diagnosed with paraphilia. Individuals with substance abuse problems referred to PT have a shorter LoS. They are discharged even if their societal risk is relatively higher than that of people with psychotic diagnoses, which is reflected in higher HoNOS-secure score. We failed to find a relationship between crime severity and LoS. This can be interpreted as an indication that PT is functioning properly because it provides treatment for mental conditions rather than punishment for the crime committed. If the patient’s health condition is stable or assured, the patient is conditionally released regardless of the severity of the index offense.

However, we are cautious about generalizing the results of this study to the entire Czech PT population. We cannot exclude differences in the diagnostic spectrum, treatment regimen, and legal procedures between forensic facilities, possibly limiting the generalizability of the results and justifying further studies that enable benchmarking of various forensic facilities.

## Data Availability Statement

The raw data supporting the conclusions of this article will be made available by the authors, without undue reservation.

## Ethics Statement

The studies involving human participants were reviewed and approved by the Ethics Committee of Psychiatric Hospital Bohnice, Prague, Czechia. Written informed consent for participation was not required for this study in accordance with the national legislation and the institutional requirements.

## Author Contributions

MP: conception and design of the project, analysis and interpretation of data, and manuscript drafting and revision. IS: analysis and interpretation of data and manuscript drafting and revision. MV: analysis and interpretation of data, manuscript drafting and revision, and final preparation of the manuscript. All authors contributed to the article and approved the submitted version.

## Conflict of Interest

The authors declare that the research was conducted in the absence of any commercial or financial relationships that could be construed as a potential conflict of interest.

## Publisher’s Note

All claims expressed in this article are solely those of the authors and do not necessarily represent those of their affiliated organizations, or those of the publisher, the editors and the reviewers. Any product that may be evaluated in this article, or claim that may be made by its manufacturer, is not guaranteed or endorsed by the publisher.
