# Supplementary material for: Factors Affecting Length of Inpatient Forensic Stay: Retrospective Study From Czechia
Source: Front Psychiatry. 2022 May 4;13:825615. doi: 10.3389/fpsyt.2022.825615 (PMC9114463; doi:10.3389/fpsyt.2022.825615)
Supplement: Supplementary file 1 [file Table_1.DOCX]

**APPENDIX I**

| New Groupings | | | | | | |
| --- | --- | --- | --- | --- | --- | --- |
| Age | | | | | | |
| R^2^ = 0.048, Adjusted R^2^= 0.044, F(258, 1) = 13.15, p = <.001 | | | | | | |
|  | Estimate | Std. Error | t-value | *p* value | 95% CI | |
| (Intercept) | -12.73 | 146.53 | -0.08 | 0.930 | [-599.17, 239.28] | |
| age | 12.04 | 3.32 | 3.62 | 0.001 | [5.59, 28.02] | |
| Sex (0 male, 1 ,female) | | | | | | |
| R^2^= 0.018, Adjusted R^2^= 0.014, F(258, 1) = 4.72, *p* = 0.031 | | | | | | |
|  | Estimate | Std. Error | t-value | *p* value | 95% CI | |
| (Intercept) | 531.7 | 46.06 | 11.54 | <.001 | [453.5, 652.3] | |
| sex | -281.15 | 129.29 | -2.17 | 0.030 | [-401.3, -189.9] | |
| Marital Status Binary | | | | | | |
| R^2^= 0.020, Adjusted R^2^= 0.016, F(257, 1) = 5.24, *p* = 0.022 | | | | | | |
|  | Estimate | Std. Error | t-value | *p* value | 95% CI | |
| Not married or engaged (Intercept) | 556.82 | 50.79 | 10.96 | <.001 | [464.3, 700.7] | |
| Married or Engaged | -220.68 | 96.33 | -2.29 | 0.022 | [-368.4, -99.2] | |
| Education | | | | | | |
| R^2^= 0.004, Adjusted R^2^= -0.003, F(242, 2) = 0.51, *p* = 0.596 | | | | | | |
|  | Estimate | Std. Error | t-value | *p* value | 95% CI | |
| basic education (reference) | 513.09 | 48.18 | 10.64 | <.001 | [427.8, 642.3] | |
| Graduation | -52.44 | 97 | -0.54 | 0.589 | [-201.24, 125.1] | |
| High education | -146.09 | 156.12 | -0.93 | 0.35 | [-302.2, 38.2] | |
| Employment | | | | | | |
| R^2^= 0.026, Adjusted R^2^= 0.014, F(252, 3) = 2.25, *p* = 0.082 | | | | | | |
|  | Estimate | Std. Error | t-value | *p* value | 95% CI | |
| Unemployed (reference) | 558.63 | 56.67 | 9.857 | <.001 | [446.9, 740.1] | |
| Stable Employment | -266.11 | 108.41 | -2.455 | 0.014 | [-448.0, -140.4] | |
| Part time | -24.34 | 115.62 | -0.21 | 0.833 | [-230.50, 188.23] | |
| Other/Prison | -132.12 | 98.44 | -1.342 | 0.180 | [-327.2, 62.2] | |
| Housing | | | | | | |
| R^2^= 0.00016, Adjusted R^2^= -0.0037, F(253, 1) = 0.040, *p* =.839 | | | | | | |
|  | Estimate | Std. Error | t-value | *p* value | 95% CI | |
| Other (Intercept) | 485.42 | 48.13 | 10.087 | <.001 | [409.6, 600.1] | |
| Any housing | -16.47 | 81.46 | -0.202 | 0.84 | [-160.37, 163.26] | |
| PT Type(1 as reference) | | | | | | |
| R^2^= 0.045, Adjusted R^2^= 0.034, F(256, 3) = 4.11, *p* = 0.007 | | | | | | |
|  | Estimate | Std. Error | t-value | P value | 95% CI | |
| Psychiatric (Intercept) | 593.69 | 59.54 | 9.971 | <.001 | [475.5, 788.4] | |
| Sex offender | 160.36 | 164.69 | 0.974 | 0.331 | [-127.2, 524.4] | |
| Combination | -244.82 | 98.33 | -2.49 | 0.013 | [-440.7, -94.3] | |
| Substance abuse | -325.09 | 138.79 | -2.342 | 0.019 | [-521.7, -189.6] | |
| Before Treatment | | | | | | |
| R^2^= 0.017, Adjusted R^2^= -0.002, F(254, 5) = 0.88, *p* = 0.49 | | | | | | |
|  | Estimate | Std. Error | t-value | *p* value | 95% CI |  |
| Patient was at home (reference) | 529.67 | 66.12 | 8.011 | <.001 | [411.8, 737.8] | |
| Prison | -131.56 | 102.43 | -1.284 | 0.2 | [-334.5, 33.8] | |
| Secure detention | 178.83 | 356.07 | 0.502 | 0.61 | [-266.1, 1325] | |
| Hospitalized at different ward | -109.67 | 145.79 | -0.752 | 0.45 | [-323.4, 55.9] | |
| Outpatient PT | 136.3 | 140.26 | 0.972 | 0.33 | [-153.8, 592.3] | |
| Other | -41 | 409.37 | -0.1 | 0.92 | [-378.80, 437.15] | |
| Previous Hospitalisations | | | | | | |
| R^2^= 0.040, Adjusted R^2^= 0.033, F(255, 2) = 5.41, *p* = 0.004 | | | | | | |
|  | Estimate | Std. Error | t-value | *p* value | 95% CI | |
| (Intercept) | 450.2217 | 47.5793 | 9.463 | <.001 | [386.2, 549.6] | |
| Previous Hospitalisation Count | -26.165 | 14.7235 | -1.777 | 0.076 | [-66.97, 2.87] | |
| Previous Hospitalisation Length (days) | 1.2275 | 0.4141 | 2.964 | 0.003 | [0.263, 2.838] | |
| Disorders | | | | | | |
| R^2^= 0.122, Adjusted R^2^= 0.090, F(250, 9) = 3.86, *p* = 0.001 | | | | | | |
|  | Estimate | Std. Error | t-value | *p* value | 95% CI | |
| (Intercept) | 215.92 | 120.31 | 1.795 | 0.073 | [-24.8, 556.4] | |
| Physio. Dis. | 139.04 | 185.6 | 0.749 | 0.454 | [-103.0, 479.6] | |
| Substance Dis. | 22.71 | 103.76 | 0.219 | 0.826 | [-312.33, 224.98] | |
| Psychotic | 391.72 | 98.64 | 3.971 | <.001 | [208.4, 700.3] | |
| Mood Dis. | 176.68 | 233.2 | 0.758 | 0.449 | [-91.6, 966.5] | |
| Stress Dis. | 149.16 | 303.63 | 0.491 | 0.623 | [-244.3, 922.1] | |
| Personality Disorders (para) | 488.49 | 141.8 | 3.445 | 0.000 | [249.2, 816.6] | |
| Personality Disorders (F60.62) | 89.66 | 91.66 | 0.978 | 0.328 | [-53.89, 325.57] | |
| Intel. Dis. | 878.12 | 278.76 | 3.15 | 0.001 | [-77.4, 2847.2] | |
| Develop. Dis | 253.33 | 486.18 | 0.521 | 0.602 | [-82.9, 566.4] | |
| Medication and Resistance | | | | | | |
| R^2^= 0.133, Adjusted R^2^= 0.112, F(253, 6) = 6.47 , *p* = 0.001 | | | | | | |
|  | Estimate | Std. Error | t-value | *p* value | 95% CI | |
| (Intercept) | 324.06 | 66.36 | 4.884 | <.001 | [264.4, 392.5] | |
| Antipsychotics | 116.79 | 91.42 | 1.278 | 0.202 | [7.4, 259.5] | |
| Anti-Depress. | 178.93 | 95.64 | 1.871 | 0.062 | [3.4, 410.1] | |
| Anxiolytic | -261.97 | 226.99 | -1.154 | 0.249 | [-600.8, 246.7] | |
| Thymostabilisers | -186.76 | 111.14 | -1.681 | 0.094 | [-449.0, 67.9] | |
| Antiandrogenes | 278.99 | 178.72 | 1.561 | 0.119 | [-1.9, 670.1] | |
| Pharmacoresistance | 653.14 | 127.27 | 5.132 | <.001 | [245.3, 1278.9] | |
| Regular Support | | | | | | |
| R^2^= 0.018, Adjusted R^2^= 0.011, F(254, 2) = 2.438, *p* = 0.089 | | | | | | |
|  | Estimate | Std. Error | t-value | *p* value | 95% CI | |
| Friends(intercept) | 470.77 | 49.16 | 9.577 | <.001 | [399.2, 590.7] | |
| Family | -67.6 | 150.85 | -0.448 | 0.654 | [-217.31, 109.09] | |
| None | 282.14 | 134.77 | 2.093 | 0.037 | [-5.2, 753.7] | |
| Housing Option After | | | | | | |
| R^2^= 0.209, Adjusted R^2^= 0.200, F(256, 3) = 22.6, *p* = 0.001 | | | | | | |
|  | Estimate | Std. Error | t-value | *p* value | 95% CI | |
| Falt (Intercept) | 339.1 | 52.27 | 6.487 | <.001 | [296.4, 404.7] | |
| Missing or no housing | 916.98 | 115.3 | 7.953 | <.001 | [577.6, 1462.2] | |
| Special housing | 196.6 | 107.77 | 1.824 | 0.069 | [23.1, 540] | |
| Hostel or rent | -49.52 | 116.56 | -0.425 | 0.671 | [-136.67, 84.28] | |
| Change BI | | | | | | |
| R^2^= 0.003, Adjusted R^2^= -0.0005, F(255, 1) =.853, *p* = 0.356 | | | | | | |
|  | Estimate | Std. Error | t-value | *p* value | 95% CI | |
| (Intercept) | 447.08 | 69.62 | 6.422 | <.001 | [344.7, 680.9] | |
| Change to outpatient treatment or termination of treatment[DI] Binary (1 to outpatient, 0 - other) | 82.83 | 89.65 | 0.924 | 0.35 | [-138.92, 236.51] | |
| Change 3 groups | | | | | | |
| R = 0.008,Adjusted R = 0.001, F(254, 2) = 1.136, *p* = 0.322 | | | | | | |
|  | Estimate | Std. Error | t-value | *p* value | 95% CI | |
| (Intercept) (other options) | 268.2 | 165.6 | 1.619 | 0.107 | [208.3, 348.1] | |
| Conditional discharge | 261.7 | 174.9 | 1.496 | 0.136 | [147.3, 398.2] | |
| Treatment termination | 217.2 | 182.5 | 1.191 | 0.235 | [76.6, 504.2] | |
| Change 2 new groups | | | | | | |
| R^2^= 0.008, Adjusted R^2^= 0.004, F(255, 1) = 2.06, *p* = 0.152 | | | | | | |
|  | Estimate | Std. Error | t-value | *p* value | 95% CI | |
| (Intercept) | 268.2 | 165.3 | 1.622 | 0.106 | [208.3, 348.1] | |
| Conditional discharge or Treatment termination | 246.1 | 171.5 | 1.435 | 0.152 | [140.5, 370.5] | |
| Crime Single item | | | | | | |
| R^2^= 0.039,Adjusted R^2^= 0.020, F(254, 5) = 2.083, *p* = 0.068 | | | | | | |
|  | Estimate | Std. Error | t-value | *p* value | 95% CI | |
| (Intercept) | 592.65 | 56.94 | 10.408 | <.001 | [483.1, 751.9] | |
| Substance possession (0-no, 1-yes) | -155.63 | 147.44 | -1.056 | 0.292 | [-288.4, -6.6] | |
| Obstruction of Execution of legal decision (0-no, 1-yes) | -201.4 | 138.61 | -1.453 | 0.147 | [-351.3, -7.3] | |
| Arson (0-no, 1-yes) | 924.74 | 497.67 | 1.858 | 0.064 | [107, 1777] | |
| Spreading Alarm (0-no, 1-yes) | 30.88 | 402.28 | 0.077 | 0.938 | [-103.74, 162.26] | |
| Crime Committed Under Substance(s) (0-no, 1-yes) | -188.57 | 90.57 | -2.082 | 0.038 | [-334.2, -33.3] | |
| Composite Crime | | | | | | |
| R^2^= 0.022, Adjusted R^2^= 0.011, F(256, 3) = 1.968, *p* = 0.119 | | | | | | |
|  | Estimate | Std. Error | t-value | *p* value | 95% CI | |
| (Intercept) | 504.745 | 88.733 | 5.688 | <.001 | [394.5, 666.3] | |
| At least one violent Crime (0-no, 1-yes) | -63.625 | 96.03 | -0.663 | 0.508 | [-235.95, 86.21] | |
| At least one sexual crime (0-no, 1-yes) | 299.206 | 141.943 | 2.108 | 0.036 | [62.8, 665.6] | |
| At least one property crime (0-no, 1-yes) | -1.359 | 113.788 | -0.012 | 0.99 | [-162.162, 246.673] | |
| Ola Equiv.(Antispychotics) | | | | | | |
| R = 0.068,Adjusted R = 0.064, F(249, 1) = 18.28, *p* = 0.001 | | | | | | |
|  | Estimate | Std. Error | t-value | *p* value | 95% CI | |
| (Intercept) | 349.545 | 46.397 | 7.534 | <.001 | [278.9, 419.2] | |
| Total Olanzapine Equivalent | 11.318 | 2.647 | 4.276 | <.001 | [4.83, 23.12] | |
| Ward Type DL item | | | | | | |
| R^2^= 0.045, Adjusted R^2^= 0.037, F(257, 2) = 6.06, *p* = 0.002 | | | | | | |
|  | Estimate | Std. Error | t-value | *p* value | 95% CI | |
| Medium Security(intercept) | 378 | 54.9 | 6.886 | <.001 | [327.3, 451.9] | |
| Open | 347.3 | 110.6 | 3.14 | 0.001 | [161.1, 661.9] | |
| Low Security | 244.6 | 109 | 2.244 | 0.025 | [14.3, 671.1] | |
| Ward Grouping (medium) | | | | | | |
| R^2^= 0.039, Adjusted R^2^= 0.035, F(258, 1) = 10.55, *p* = 0.001 | | | | | | |
|  | Estimate | Std. Error | t-value | *p* value | 95% CI | |
| (Intercept) | 662.71 | 66.67 | 9.94 | <.001 | [515.4, 900.7] | |
| Ward Grouping (medium) | -281.43 | 86.63 | -3.249 | 0.001 | [-519.9, -118.3] | |
| HONOS | | | | | | |
| R^2^= 0.1217, Adjusted R^2^= 0.1149, F(257, 2) = 17.8, *p* < 0.001 | | | | | | |
|  | Estimate | Std. Error | t-value | *p* value | 95% CI | |
| (Intercept) | 620.88 | 79.15 | 7.845 | <.001 | [483.9, 793.4] | |
| HONOS Secure Total | -109.25 | 25.84 | -4.227 | <.001 | [-177.8, -63.6] | |
| HONOS Total | 100.6 | 17.45 | 5.765 | <.001 | [48.2, 208.6] | |
| Dysfunction | | | | | | |
| R = 0.039,Adjusted R = 0.035, F(248, 1) = 10.18, *p* = 0.001 |  | | | | | |
|  | Estimate | Std. Error | t-value | *p* value | 95% CI | |
| (Intercept) | 362.34 | 54.57 | 6.64 | <.001 | [286.3, 453.2] | |
| Psychological Dysfunction | 112.29 | 35.19 | 3.191 | 0.001 | [37.7, 221.6] | |
